# Structure fragmentation in Fe-based alloys by means of cyclic martensitic transformations of different types

**DOI:** 10.1186/1556-276X-9-92

**Published:** 2014-02-24

**Authors:** Volodimir I Bondar, Vitalij Ie Danilchenko, Ievgenij M Dzevin

**Affiliations:** 1 G.V.Kurdyumov Institute for Metal Physics, NAS of Ukraine, Vernadsky blvd. 36, Kiev 03680, Ukraine

**Keywords:** Thermocycling, Austenite, Martensite transformation, Ultradispersive and nanocrystalline structure

## Abstract

The effect of martensite transformations of different types on the misorientation of austenite crystalline lattice, which characterizes the degree of structure fragmentation, was investigated for Fe-Ni and Fe-Mn alloys. As a result of multiple face-centered cubic (f.c.c.)-body-centered cubic (b.c.c.)-f.c.c. transformations, an austenite single-crystalline specimen is transformed in a polycrystalline one due to progressive fragmentation. It was shown that the degree of fragmentation depends on the magnitude of volume change and the density of dislocations generated on martensitic transformations.

## Background

Methods of producing nanostructured materials such as powder metallurgy, inert gas condensation, mechanical milling, melt quenching, or crystallization of an amorphous material have received much attention [1,2]. Another approach for the preparation of highly dispersive materials is cyclic plastic deformation, which is viable for particular classes of metallic materials.

The crystallographic orientation of initial austenite in Fe-based alloys is nonideally restored after reverse martensite transformation [3]. This is associated with the incomplete reversibility of direct and reverse martensite transformations and an accumulation of dislocations in the reverted austenite [4,5]. Formation of structure defects on martensite transformations results in azimuthal tailing of diffraction reflections of single-crystalline samples. From the magnitude of tailing and from the increase of the misorientation angle of crystal lattice regions after multiple martensitic transformations, one can deduce the capability of fragmentation and grain refinement of an austenite phase [4,6].

In Fe-based alloys, three types of martensitic transformations are realized: γ-α-γ in Fe-Ni-based alloys with face-centered cubic (f.c.c.)-body-centered cubic (b.c.c.)-f.c.c. structure rebuilding, γ-ϵ-γ in Fe-Mn-based alloys with f.c.c.-hexagonal close-packed (h.c.p.)-f.c.c. transformation [7], and γ-ϵ′-γ in Fe-Mn-based alloys with f.c.c.-18-layer rhombic (18R)-f.c.c. transformation [8,9]. It is shown experimentally that the restoration of the initial austenite structure after cyclic γ-ϵ-γ and γ-ϵ′-γ transformations turned out to be superior against that of alloys with γ-α-γ transformations. This regularity is based on the fact that the density of dislocations increases by more than 10^3^ after cyclic γ-α-γ transformations connected with a high volume change - up to 3% to 4%, while it increases only by 10 after cyclic γ-ϵ-γ transformations (with a smaller volume change - up to approximately 0.75%) and practically does not change after γ-ϵ′-γ transformations (volume change - up to approximately 0.5%) [4,7]. In the austenitic phase, additional subgrain boundaries can form under conditions of dislocation generation by direct and reverse martensite transformations, for example, by means of wall formation by one-sign dislocations. On account of these processes, the fragmented structure of reverted austenite is received. The process of structure fragmentation can be essentially different for alloys with different types of martensitic transformations.

In the present article, the effect of multiple martensitic transformations of different types is studied in Fe-Ni- and Fe-Mn-type alloys. The development of austenitic structure fragmentation and the capability of particular alloys to form highly dispersive structures due to the accumulation of structure defects are elucidated.

## Methods

The following alloys: Fe - 24.8 wt.%, Ni - 0.50 wt.%, C (alloy 1); Fe - 19.5 wt.%, Mn - 2 wt.%, Si (alloy 2); Fe - 16.7 wt.%, Mn - 0.45 wt.%, C (alloy 3), and Fe - 15.2 wt.%, Mn - 0.32 wt.%, C (alloy 4), were chosen for the investigation. All the alloys were melted in a furnace in purified argon. Single-crystalline samples (∅ 0.8 mm, *L* = 5 to 10 mm in size) for X-ray investigations in an RKV-86 (Moscow, USSR) rotational camera were cut out from large grains of the bar. All the alloys display an austenitic structure at room temperature after quenching from 1,000°C to 1,050°C in cold water. Direct γ-α, γ-ϵ, and γ-ϵ′ martensitic transformations took place during cooling in liquid nitrogen but reverse α-γ, ϵ-γ, and ϵ′-γ transformations during heating of quenched alloys in a saline bath at the rate of 60 K/s. The maximum misorientation angle *ψ* of the crystal lattice, which characterizes the degree of structure fragmentation, was found from azimuthal tailing of diffraction reflections compared to single-crystalline samples. The sizes of fragments were measured by electron microscopy (microscope PREM-200, Moscow, USSR).

## Results and discussion

### γ-α-γ transformations

**Figure 1:**
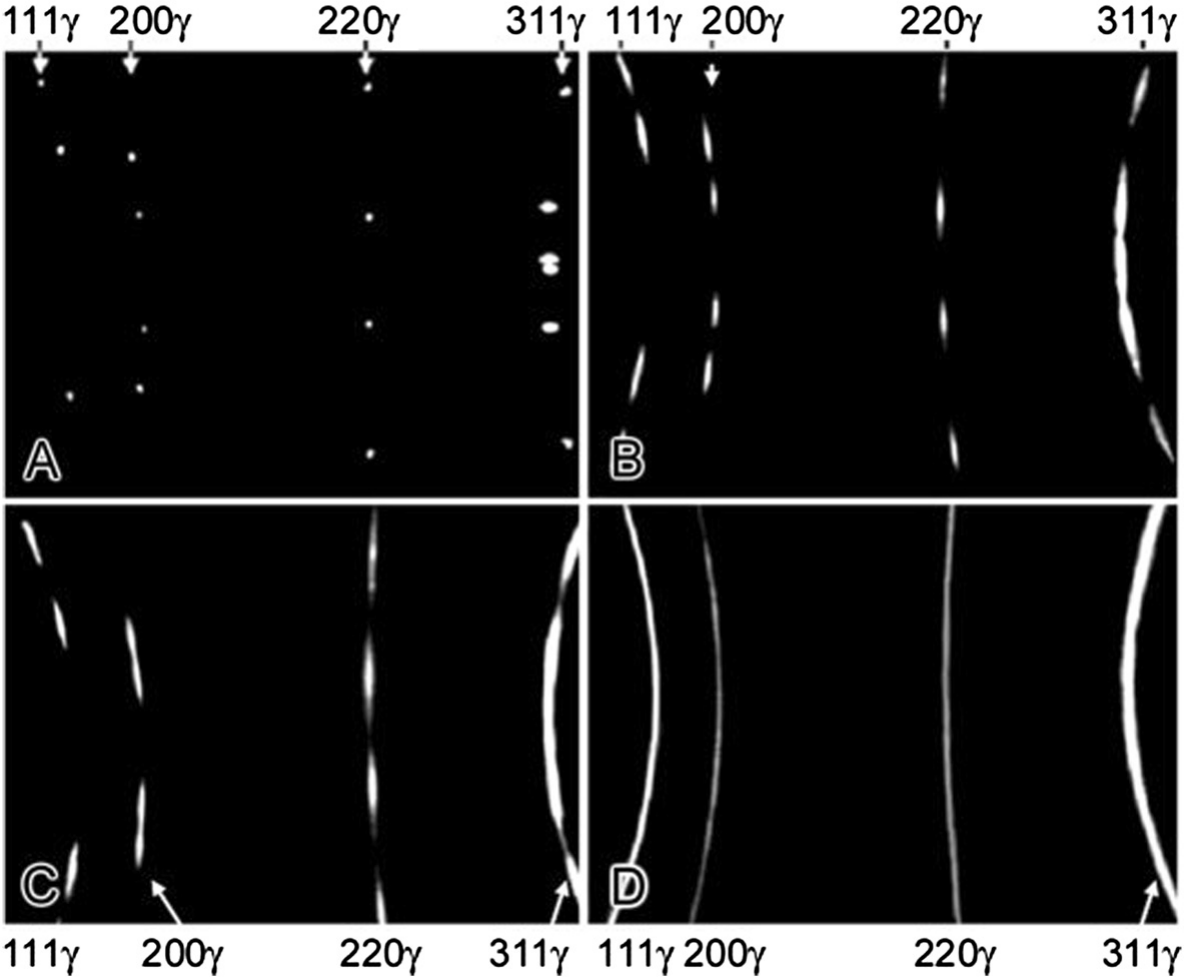
**X-ray patterns of alloy 1 single crystal in the austenitic state (f.c.c.), FeK**_**α **_**radiation.** Initial state **(A)** and after 1 **(B)**, 10 **(C)**, and 80 **(D)** γ-α-γ transformations.

**Figure 2:**
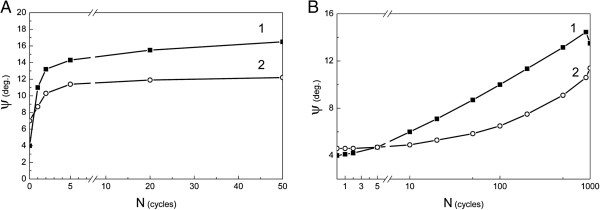
**Misorientation angle *ψ *of austenite (1) and martensite (2) in alloys 1 (A) and 2 (B).***N*, number of thermocycles.

X-ray studies of alloy 1 have shown that all the austenitic reflections present in the single-crystalline samples are washed out in the azimuthal direction after reverse α-γ transformation. On the pole figure (homostereographic projection), the centers of all initial and reversed austenitic reflections coincided at the region of measurement accuracy (1° to 2°). Azimuthal tailing of reflections monotonously increased with the increase of the quantity of transformation cycles (Figure 1A,B,C,D). At the same time, the angle *ψ* of martensite was always less than that of austenite (Figure 2A,B). Debye lines on the X-ray pattern filled up in the azimuthal direction. Hence, the rotational X-ray pattern of single-crystalline samples after 35 to 50 γ-α-γ transformations was the same as that of a textured polycrystalline sample. After 80 to 120 γ-α-γ cycles, the diffraction pattern displays practically continuous lines of austenite. It indicates a practically full recrystallization of austenite and a transformation of the initial single crystalline into a polycrystalline sample. Different azimuthal tailing of the γ and α phase reflections qualify the different degrees of crystal lattice fragmentation of the austenite and the martensite phase, respectively.

Electron microscopic investigation has shown that in the process of thermocycling, subgrain boundaries were created in reversed austenite. These boundaries were formed by dislocations generated by repeated γ-α and α-γ transformations. At a certain stage, subgrain boundaries form the observed fragments in the initial austenite grains. After 10 to 20 cycles, the decomposition of the reflections into three to five components was observed (Figure 1B) parallel with the progress of azimuthal tailing of reflections on the electron diffraction pattern that provide evidence for the formation of additional subgrain boundaries at this stage. In reversed austenite, the fragment size decreased with increasing number of transformation cycles. After 30 cycles, the major fraction of fragments was in the range 0.2 to 0.8 μm. After 80 to 100 cycles, the size of fragments reached the nanoscale level (about 100 nm). The structure of the boundaries of fragments exhibits a dislocation nature analogous to other grain boundaries after intensive plastic deformation [10-12].

Multiple thermocycling (>30 cycles) forced the formation of reversed austenitic twins. The volume content of twins increased with the increasing number of γ-α-γ transformations owing to the accumulation of internal stresses in the γ phase.

Thus, the multiple thermocycling of alloy 1 with ongoing direct γ-α and reverse α-γ martensite transformations led to fragmentation of the initial austenite up to a nanoscale level (nanofragmentation).

### γ-ϵ-γ transformations

The X-ray investigations of the iron-manganese single-crystalline samples of alloy 2 have shown that the initial orientation is nonideally restored as in the case of the iron-nickel alloys (alloy 1). However, it is considerably better recovered after γ-ϵ-γ transformations in Fe-Mn alloys than after γ-α-γ transformations in Fe-Ni alloys.

The X-ray rotational and rocking patterns of the thermally cycled austenite of alloy 2 show the tailing of all diffraction reflections of the γ and ϵ phases (Figure 1B). The misorientation angle increases much less than that for γ-α-γ transformations. Thus, after the first γ-ϵ-γ cycle, *ψ* = 4°, but after 150 cycles, *ψ* = 10° is reached, a magnitude which is almost achieved after the first γ-α-γ transformation in alloy 1.

However, full recrystallization of the austenite of alloy 2 was not achieved by repeated γ-ϵ-γ transformations, and even after 1,000 cycles, only *ψ* ≤ 17° was realized and the Debye lines on the X-ray patterns were not really continuous. It is important to note that the initial orientation of the austenite single-crystalline sample was restored and no new orientations appeared in this case.

Misorientation as a result of precipitation hardening in the α-martensite of alloy 1 is much higher than that in the ϵ-martensite of alloy 2, although for both cases, it is smaller than that for the corresponding austenite phases.

### γ-ϵ′-γ transformations

**Figure 3:**
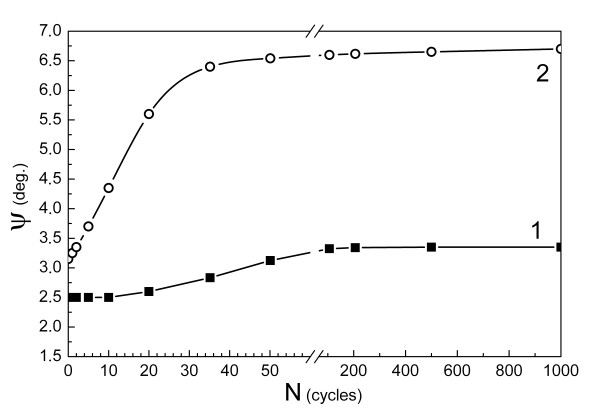
**Misorientation angle *ψ *of the austenite of alloys 3 (1) and 4 (2).***N*, number of thermocycles.

The initial orientation of the austenite of alloy 3 was completely restored during these transformations. Azimuthal tailing of the diffraction reflections of austenite increased only slightly with the number of cycles (Figure 3, curve 1), and even after 1,000 cycles, only *ψ* ≤ 3.5° was reached.

In alloy 4, where the γ-ϵ transformation was observed during quenching, the quantity of ϵ-martensite is diminished during -196°C ↔ 300°C cycles, and only γ-ϵ′-γ transformations are taking place after 20 to 30 cycles.

Azimuthal tailing of the γ-phase reflections was observed mainly within the first 10 cycles, during which γ-ϵ-γ transformations proceed (Figure 3). The misorientation of austenite did not change during the subsequent γ-ϵ′-γ transformations. Thus, misorientation is constrained to *ψ* ≤ 7° if the number of γ-ϵ′-γ transformations increased from 20 to 1,000, whereas *ψ* = 6° is already achieved after the first 10 cycles.

**Figure 4:**
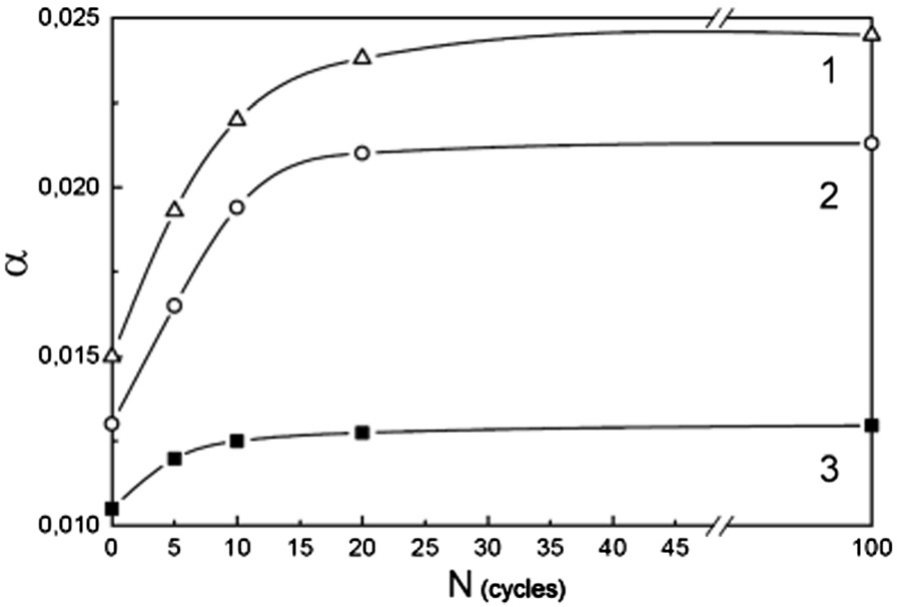
**Concentration of chaotic packing defects α as a function of the number of thermocycles *N*.** 1 - alloy 2, 2 - alloy 3, 3 - alloy 4.

A great quantity of chaotic packing defects (CPD) are generated in austenite during the process of γ-ϵ-γ and γ-ϵ′-γ martensitic transformations [7,13]. The role of CPD in the formation of additional subboundaries is not investigated here. In this connection, the change of CPD concentration at multiple martensitic transformations has been studied for the Fe-Mn-based alloys 2, 3, and 4. The concentration of CPD was measured by the relative displacement of austenitic (111)γ and (222)γ reflections [14,15]. It is apparent that the concentration of CPD in alloy 3 (forming ϵ′-martensite) does not exceed 0.015 (Figure 4). In this alloy, the austenitic lattice misorientation is insignificant and not accumulated for multiple γ-ϵ′-γ transformations (Figure 3). This means that a small CPD concentration does not lead to the formation of additional subgrain boundaries and to the fragmentation of reversed austenite. In alloys 3 and 4, the concentration of CPD exceeds the magnitudes 0.022 and 0.025, respectively (Figure 4) and austenitic lattice misorientation reached 17° and 6.5°, respectively (Figures 1 and 3). Obviously, starting from this CPD concentration, the disoriented fragments form in the microstructure of reversed austenite. These results show that with the increase of CPD concentration in austenite, the ability to form disoriented fragments of its lattice increases.

## Conclusions

The γ-ϵ-γ and γ-ϵ′-γ transformations in iron-manganese alloys resulted in a smaller increase of the misorientation angle *ψ* than that for γ-α-γ transformations in the iron-nickel alloys. This is due to the smaller number of crystal structure defects generated by γ-ϵ-γ transformations. In fact, the dislocation density of the austenite increases by 3 orders of magnitude after the γ-α-γ transformation, but it is constrained to less than 1 order of magnitude after the γ-ϵ-γ transformation. The misorientation is changed to a still smaller amount during γ-ϵ′-γ transformations.

Thus, the sequence of the magnitude of the misorientation angle *ψ* during martensitic transformations in iron-based alloys can be described as

$$\psi_{\gamma ‒\alpha ‒\gamma }>\psi_{\gamma ‒\epsilon ‒\gamma }>\psi_{\gamma ‒\epsilon^{\prime }‒\epsilon }$$

Accumulation of the dislocations at multiple f.c.c.-b.c.c.-f.c.c. martensite transformations in iron-nickel alloys led to full recrystallization of austenite due to the formation of lattice fragments with significant mutual misorientation and to a transformation of the single-crystalline sample into a polycrystalline one.

Multiple f.c.c.-h.c.p.-f.c.c. martensite transformations in iron-manganese alloys, on the other hand, led to the formation of additional subgrain boundaries in austenite by accumulation of CPD up to a magnitude exceeding 0.02. A full recrystallization of austenite at multiple f.c.c.-h.c.p.-f.c.c. and f.c.c.-18R-f.c.c. transformations was never observed.

## Competing interests

The authors declare that they have no competing interests.

## Authors' contributions

VB has made the main idea of investigation, acquisition, and interpretation of X-ray data and has been involved in drafting the manuscript. VD is accountable for all aspects of the work and critically revised the manuscript for important intellectual content. ID has prepared all the alloys and specimens, took part in the acquisition and interpretation of data, has been involved in drafting the manuscript, and has given final approval of the version to be published. All authors read and approved the final manuscript.
